# Large Cavitary Lung Lesions in a Patient with Severe COVID-19 Pneumonia

**DOI:** 10.4269/ajtmh.21-0949

**Published:** 2021-10-20

**Authors:** Sergey Avdeev, Galia Nuralieva, Galina Nekludova

**Affiliations:** ^1^ Sechenov First Moscow State Medical University (Sechenov University), Moscow, Russia;; ^2^ Pulmonology Scientific Research Institute, Federal Medical and Biological Agency of Russian Federation, Moscow, Russia

**Figure 1.**
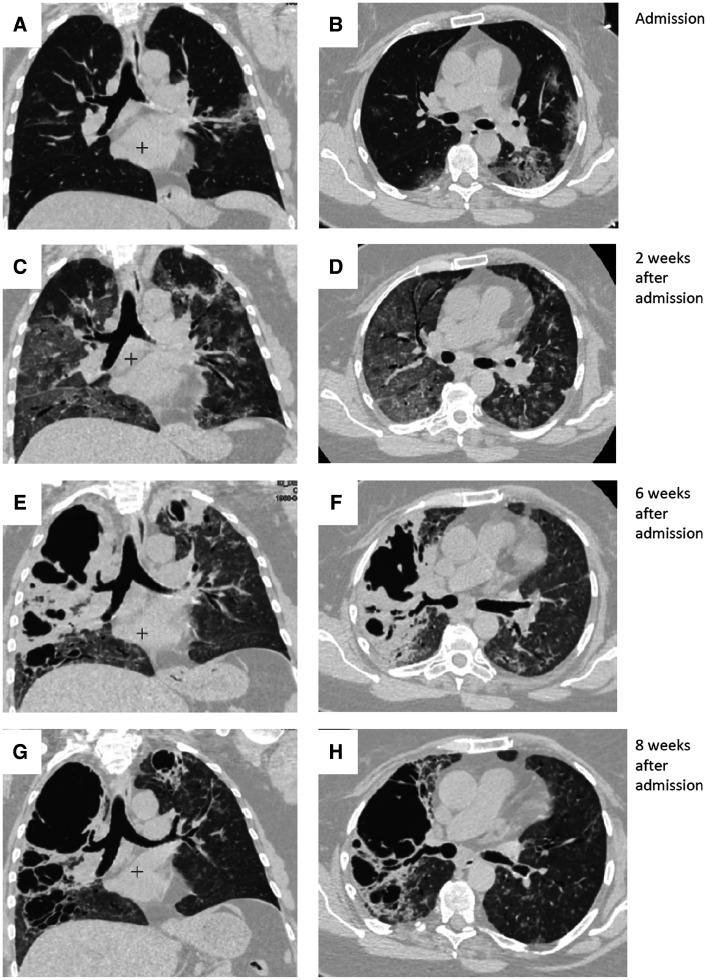
Thoracic computed tomographic scans (**A, B**) at admission showing diffuse bilateral ground-glass opacities predominantly involving the left lower lobe; (**C, D**) 2 weeks after admission showing diffuse subtotal bilateral ground-glass attenuation and linear opacities; (**E, F**) 6 weeks after admission showing extensive bilateral thick-walled, irregularly shaped cavities, with several coalescing into a larger one; and (**G, H**) 8 weeks after admission showing persistent bilateral cavitary lesions. (**A, C, E, G**) Coronal views. (**B, D, F, H**) Axial views below the carina.

A 63-year-old female non-smoker with a history of hypertension and chronic kidney disease was admitted to our hospital for dyspnea, fever (39.1°C), and myalgia. Coronavirus disease 2019 (COVID-19) infection was confirmed by positive detection of severe acute respiratory syndrome coronavirus 2 RNA from a nasopharyngeal swab. At admission, the chest computed tomography (CT) scan showed bilateral, patchy ground-glass opacities (Figure 1A and B). The patient was treated with dexamethasone (6 mg/day), enoxaparin (prophylactic dose), and tocilizumab (800 mg intravenously). One week after admission, desaturation (75% on ambient air) and tachypnea (34 breaths/min) developed. A CT scan performed at that time showed subtotal bilateral ground-glass attenuation (Figure 1C and D). The patient was treated with non-invasive ventilation (continuous positive airway pressure, 8–12 cmH_2_O, with a fraction of inspired oxygen of 60–90%) and, after 4 weeks of continuous positive airway pressure, therapy was weaned successfully to mask oxygen.

One week later, the patient developed fever and had increased levels of C-reactive protein (175 U/mL) and white blood cells (15.3 × 10^9^ cells/L). Chest CT scan showed extensive bilateral, thick-walled cavities, with several coalescing into a larger one (Figure 1E and F). Empiric antibacterial therapy was started and lasted for 2 weeks (amoxicillin–clavulanic acid and levofloxacin, followed with moxifloxacin). Our patient did not receive empiric antifungal coverage. Culture of multiple respiratory specimens showed only *Staphylococcus haemolyticus* in a low titer in one sample, and culture on Sabouraud agar remained sterile. The procalcitonin levels were low in all samples performed (≤ 0.1 ng/mL). Polymerase chain reaction assay for *Aspergillus* was negative in two sputum samples. Serum galactomannan was performed twice, but showed low levels (≤ 0.2 µg/L). Acid-fast bacilli were negative on smear, so *Mycobacterium tuberculosis* infection was not identified. Repeated blood cultures also showed no growth. The patient was managed conservatively, all inflammatory markers decreased significantly, and a CT scan 8 weeks after admission did not change significantly (Figure 1G and H). The patient was discharged home with continuous oxygen at 5 L/min.

Pulmonary cavitary lesions are rare manifestations of COVID-19 pneumonia.^1^ According to one case series, the overall prevalence of this complication is ≈3% of patients who developed COVID-19 pneumonia.^2^ The mechanisms of pulmonary cavitary lesions in COVID-19 are still unknown. Possible causes of cavitation are bacterial, fungal, or mycobacterial infections,^3^ but in our patient all these factors were excluded. Another plausible cause of cavitary lesions is distal pulmonary embolism or thrombosis, which can lead to pulmonary infarction.^4^^,^^5^ Pulmonary cavitation can be associated with secondary complications such as pneumothorax, hemoptysis, and superinfection; therefore, close follow-up of patients is necessary.
